# Trends in Psychiatrist-Led Care for Medicare Part B Enrollees

**DOI:** 10.1001/jamanetworkopen.2024.58160

**Published:** 2025-02-06

**Authors:** John L. Havlik, Syed Wahid, Kelsey C. Priest, Lydia Ososanya, Marissa Igunbor, Keith Humphreys

**Affiliations:** 1 Department of Psychiatry and Behavioral Sciences, Stanford University School of Medicine, Stanford, California; 2 The University of Chicago, Chicago, Illinois; 3 Medical College of Wisconsin, Milwaukee; 4 Veterans Affairs Health System Research and Development Center, Palo Alto, California

## Abstract

**Importance:**

The rising incidence of mental illness in the US underscores the need for timely access to psychiatric care, especially for vulnerable populations such as older adults and individuals with mental and physical disabilities who receive Medicare.

**Objective:**

To assess changes in the number and proportion of active psychiatrists providing professional services to traditional Medicare Part B enrollees from 2014 to 2022.

**Design, Setting, and Participants:**

This repeated cross-sectional study of Medicare Part B enrollees used publicly available data (for January 1, 2014, through December 31, 2022) from the Centers for Medicare & Medicaid Services and the Kaiser Family Foundation. Data from all traditional Medicare Part B enrollees in all 50 states and the District of Columbia were analyzed.

**Main Outcomes and Measures:**

Primary outcomes included changes in (1) the proportion of active psychiatrists billing traditional Medicare and (2) the number of psychiatrists providing care relative to the number of Medicare Part B enrollees nationally, regionally, and by state.

**Results:**

The study population comprised 291 748 472 Medicare Part B enrollee-years (33 042 936 in 2014: 5 800 903 [17.6%] eligible due to disability alone and 27 242 030 [82.4%] eligible due to age). From 2014 to 2022, the number of Medicare Part B enrollees decreased by 3 497 942 enrollee-years (10.6%), while the number of psychiatrists submitting more than 10 claims to Medicare Part B decreased by 3772 (16.8%). The nationwide proportion of professionally active psychiatrists submitting claims to Medicare Part B for professional services during this period declined from 22 409 of 50 416 (44.4%) in 2014 to 18 637 of 56 492 (33.0%) in 2022 (*P* < .001 on univariable regression). From 2014 to 2022, state-level changes in Medicare Part B–serving psychiatrists per enrollee ranged from a 31.7% increase in Alabama (from 36.5 to 48.1 psychiatrists per 100 000 enrollees) to a 67.8% decrease in Wyoming (from 42.9 to 13.8 psychiatrists per 100 000 enrollees). Every state and district assessed saw a decrease in the percentage of active psychiatrists who billed Medicare Part B for professional services over the study period.

**Conclusions and Relevance:**

In this study of Medicare Part B acceptance among active US psychiatrists, declining acceptance of Medicare Part B for professional services among psychiatrists was observed nationally and across all states. During a time of psychiatrist workforce growth, the number of psychiatrists accepting traditional Medicare decreased, indicating potential challenges in providing equitable access to psychiatric care.

## Key Points

**Question:**

How have the proportion and absolute number of psychiatrists providing professional services to traditional Medicare enrollees changed as the field has grown?

**Findings:**

In this cross-sectional study using data from 2014 to 2022, the nationwide proportion of psychiatrists billing Medicare Part B declined from 44% in 2014 to 33% in 2022. The number of psychiatrists billing Medicare Part B declined by 3772 during a time when the total number of active psychiatrists increased by 6076.

**Meaning:**

These findings suggest that during a time of psychiatrist workforce growth, the proportion and number of psychiatrists accepting Medicare Part B decreased, further suggesting potential decreases in access to psychiatrist-led care for older adults and individuals with disabilities.

## Teaser

This cross-sectional study examines trends in the number and proportion of psychiatrists providing professional services to traditional Medicare Part B enrollees from 2014 to 2022.

## Introduction

As the primary insurer of older US adults as well as an important insurer for many individuals with disabilities (including many aged <65 years with disability due to mental illness), Medicare reached 62.6 million enrollees in 2021.^1,2,3^ More than half (35.6 million) of these individuals were enrolled in traditional Medicare (in which the government pays practitioners directly on a fee-for-service model); the remainder were enrolled in Medicare Advantage (in which care is contracted by the Medicare program to private managed care organizations). Many of these older adults and individuals with disabilities have mental health and substance use disorders.^4^ Adverse outcomes from behavioral health crises are rising among older adults, with deaths due to overdose quadrupling in the past 2 decades.^5^ These adverse outcomes may be related to gaps in access to timely care; for instance, less than half (45%) of adults aged 50 years or older who needed treatment for opioid use disorder in the past year received any treatment.^6,7^ Indeed, national survey data suggest that less than 60% of office-based psychiatrists accept insurance of any kind, let alone government-sponsored insurance such as Medicare.^8,9^ This is a critical issue for Medicare, given the nontrivial price elasticity of demand for mental health services.^10^ Understanding the size of the workforce serving older adults and individuals with disabilities is critical to guide policy that may influence the behavioral health care workforce pipeline.

For decades after its creation in 1965, Medicare was not a major payer in US mental health care.^11^ Notably, traditional Medicare Part B (which covers outpatient care and many physician services during inpatient care) required enrollees to pay 50% of care costs vs only 20% for other covered services, and it also capped the annual benefit for outpatient mental health care at $250 (50% of $500).^11^ This benefit cap was raised to $1100 in 1987, and then removed entirely in 1989.^11^ Importantly, the Medicare Improvement for Patients and Providers Act of 2008 began aligning enrollees’ copay for Medicare Part B mental health coverage with that for other types of care, reaching parity (ie, 20% paid by the enrollee and 80% paid by Medicare) in 2014.^12^ Some evidence indicates this increased enrollees’ access to mental health services,^13^ but whether it has changed psychiatrists’ participation in Part B is unknown. In recent years, Medicare’s Welcome to Medicare initial examination has included screenings for depression and alcohol use disorder, which presumably might lead to more referrals to specialist care.^14^ Medicare Part B helps to pay for this visit, and nearly all other psychiatric services, for traditional Medicare enrollees seeking access to mental health care.

Although the number of professionally active psychiatrists has increased in recent years since reimbursement parity was met^15^ and the number of medical school graduates matching into psychiatry has increased for 13 consecutive years,^16^ the number and proportion of psychiatrists who choose to serve traditional Medicare enrollees has received little study. Accordingly, this repeated cross-sectional study aimed to evaluate how the number of psychiatrists providing professional services to adults on traditional Medicare Part B has changed over time, both relative to all active psychiatrists and relative to the number of adults covered by traditional Medicare Part B. Assessing psychiatrist numbers relative to the number of traditional Medicare enrollees is important because as Medicare Advantage has grown in popularity, enrollment in traditional Medicare has declined even as the program overall has expanded. To accomplish our study aims, we characterized and compared recent trends in the number of Medicare Part B enrollees and number of Medicare Part B–serving psychiatrists in the US from 2014 to 2022. We analyzed changes in the number of Medicare-serving psychiatrists and traditional Medicare enrollees nationwide, by state, and by geographic region over this period to understand variations in the growth rates of active psychiatrists, traditional Medicare enrollees, and the number of psychiatrists per Medicare enrollee. We bypassed response bias in practitioner survey data by directly examining claims filed by psychiatrists to traditional Medicare over this time period, combining 3 national datasets in an approach readily applicable by health services researchers in other specialties. This approach allowed us to assess whether the recent growth of psychiatrists entering residency^16,17^ has translated into growth in traditional Medicare Part B–accepting psychiatrists.

## Methods

### Institutional Approval and Participant Consent

This cross-sectional study was based on publicly available information in accordance with 45 CFR §46 and thus did not require institutional review board approval. Informed consent was not required because no patient-level data were collected. This study followed the Strengthening the Reporting of Observational Studies in Epidemiology (STROBE) reporting guideline.

### Data Sources and Study Population

To determine the number of psychiatrists accepting Medicare by state and year, we used the Centers for Medicare & Medicaid Services (CMS) Medicare Physician & Other Practitioners dataset files (for January 1, 2014, through December 31, 2022).^18^ This dataset includes all clinicians submitting more than 10 claims in a year for Medicare Part B with data on the location of the physician rendering services to Medicare. Medicare Part B is of particular interest because it encompasses fees from services rendered by psychiatrists across the mental health care continuum, from outpatient care to partial hospitalization programs to inpatient hospitalizations.^19^ From this dataset, we selected physicians as indicated by specialty and medical degree (MD, DO, MBBS, etc), selecting for psychiatrists using the taxonomy codes in eTable 1 in Supplement 1. We excluded physician assistants, nurse practitioners, and other advanced practice clinicians from this analysis, as well as practitioners who provided services exclusively outside the 50 US states and District of Columbia. We also excluded enrollees from outside of these areas of interest from our analysis.

To compare the numbers of psychiatrists submitting claims to traditional Medicare Part B and the number of traditional Medicare Part B enrollees in each state, we used the Medicare Monthly Enrollment database to obtain the number of traditional Medicare Part B person-years insured nationally and by state (including the District of Columbia) for the years 2014 to 2022; this person-year methodology allowed us to account for changes in a Medicare enrollee’s insurance status over time (eg, an enrollee who switched to Medicare Advantage on June 30 during a given year would count as 0.5 insured traditional Medicare Part B person-years rather than a full traditional Medicare Part B enrollee).^20^ For the purposes of this study, an insured person-year was counted as an enrollee. Supplementary eligibility data were gathered from the CMS Program Statistics–Original Medicare Enrollment dataset.^21^

### Total Active Psychiatrists

To determine a denominator for the proportion of professionally active psychiatrists accepting traditional Medicare, we queried data on professionally active psychiatrists from state-level data available on the Kaiser Family Foundation website for the years 2014 to 2022, which synthesizes active professional email and state licensing data. Data on professionally active psychiatrists for years prior to 2022 were no longer available on the Kaiser Family Foundation website at the time of our analysis; to retrieve these data, we used the Internet Archive Wayback Machine to access time- and date-stamped web crawls of these deleted pages.^22^

### Study Outcomes

First, we collated state-level data by regions identified by the US Census Bureau.^23^ For each year, we calculated the number of psychiatrists per 100 000 traditional Medicare Part B enrollees by state, region, and nationally. We further calculated the compound annual growth rate (CAGR) for psychiatrists per 100 000 Medicare enrollees for each state, region, and nationally between 2014 and 2022, alongside values for percentages of practitioner growth and Medicare population growth. We repeated these calculations by state. Finally, we calculated the proportion of active psychiatrists submitting claims to traditional Medicare Part B nationally, regionally, and by state.

### Statistical Analysis

Descriptive statistical analyses were performed on national, regional, and state levels to determine the number of psychiatrists billing traditional Medicare Part B. We reported these statistics on an absolute, per–professionally active psychiatrist, and per-enrollee basis. The significance of the trend in the number of active psychiatrists billing Medicare Part B over time was assessed using univariable linear regression. We performed additional difference-in-differences analysis to assess for any effect of statewide Medicare Advantage coverage rates at the end of our study period on the change in the proportion of statewide professionally active psychiatrists billing Medicare.

All tests were 2-tailed with significance set at *P* < .05. Stata, version 17.0 (StataCorp LLC) and Python, version 3.1 (Python Software Foundation) were used for all analyses and figure creation.

## Results

### Baseline and End Point Study Sample

Our patient study sample consisted of all traditional Medicare Part B enrollees in all 50 states and the District of Columbia, comprising 33 042 936 person-years in 2014 (5 800 903 [17.6%] eligible due to disability alone and 27 242 030 [82.4%] eligible due to age) and 29 544 994 person-years in 2022 (Table). Our physician study sample included all professionally active psychiatrists, comprising 50 416 in 2014 and 56 492 in 2022.

**Table.** Geographic Trends in Traditional Medicare Part B Enrollment and Medicare Part B–Serving Professionally Active Psychiatrists, 2014-2022

Region	Beneficiaries			Active psychiatrists		
	No. of enrollee-years		Percentage change, 2014-2022	No. of psychiatrists		Percentage change, 2014-2022
	2014	2022		2014	2022	
Midwest	7 609 269	6 455 740	−15.2	4864	3951	−18.8
Northeast	6 084 288	5 352 345	−12.0	6618	5353	−19.1
South	13 167 225	11 416 127	−13.3	6893	5812	−15.7
West	6 182 154	6 320 782	2.2	4034	3521	−12.7
National	33 042 936	29 544 994	−10.6	22 409	18 637	−16.8

### Absolute and Per-Professional Changes in Medicare Part B–Serving Psychiatrists and Medicare Enrollment

**Figure 1.**
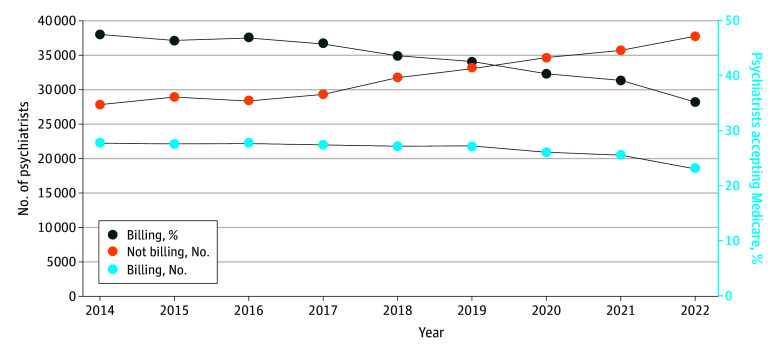
Absolute and Proportional Changes in Psychiatrists Billing and Not Billing Medicare Fee-for-Service for Professional Services, 2014-2022

From 2014 to 2022, traditional Medicare Part B enrollment declined by 10.6% (from 33 042 936 enrollee-years in 2014 to 29 544 994 enrollee-years in 2022, a net loss of 3 497 942 enrollee-years). Changes in Medicare Part B enrollment varied by geographic region over the study period (Table): Enrollment decreased 15.2% in the Midwest (from 7 609 269 enrollee-years in 2014 to 6 455 740 enrollee-years in 2022, a loss of 1 153 529 enrollee-years), 12.0% in the Northeast (from 6 084 288 to 5 352 345 enrollee-years, a loss of 731 943 enrollee-years), and 13.3% in the South (from 13 167 225 to 11 416 127 enrollee-years, a loss of 1 751 908 enrollee-years), whereas it increased by 2.2% in the West (from 6 182 154 to 6 320 782 enrollee-years, a gain of 138 628 enrollee-years). Although the absolute number of active psychiatrists increased by 12.1% nationwide from 2014 to 2022 (from 50 416 to 56 492, a net gain of 6076 psychiatrists) and increased in 48 of the 50 states and 1 federal district assessed, the nationwide number of professionally active psychiatrists submitting professional services claims to Medicare Part B declined by 16.8% (from 22 409 to 18 637 psychiatrists, a net loss of 3772 psychiatrists) nationwide over this period. This occurred alongside a decrease in the proportion of active psychiatrists submitting professional services claims to traditional Medicare Part B (from 22 409 of 50 416 active psychiatrists [44.4%] in 2014 to 18 637 of 56 492 active psychiatrists [33.0%] in 2022; β = −1.4%, *P* < .001) (Figure 1) and declined in all states and the District of Columbia. This decline in the proportion of active psychiatrists submitting claims to traditional Medicare Part B was significant even before the COVID-19 pandemic (from 22 409 of 50 416 [44.4%] in 2014 to 21 798 of 54 935 [39.7%] in 2019; β = −0.9%, *P* = .005). The absolute number of psychiatrists submitting professional services claims to Medicare Part B during this period decreased by 18.8% in the Midwest, 19.1% in the Northeast, 15.7% in the South, and 12.7% in the West.

### Per-Enrollee Changes in Medicare Part B–Serving Psychiatrists

The overall number of traditional Medicare Part B–serving psychiatrists per 100 000 Medicare enrollees decreased from 67.8 in 2014 to 63.1 in 2022 (eTable 2 in Supplement 1). In the Midwest, there were 63.9 psychiatrists per 100 000 Medicare Part B enrollees in 2014, falling to 61.2 per 100 000 enrollees in 2022. In the Northeast, there were 108.8 psychiatrists per 100 000 Medicare Part B enrollees in 2014, which decreased to 100.0 enrollees in 2022. In the South, there were 52.4 psychiatrists per 100 000 enrollees in 2014, which decreased to 50.9 per 100 000 enrollees in 2022. Finally, in the West, there were 65.3 psychiatrists per 100 000 enrollees in 2014, falling to 55.7 per 100 000 enrollees in 2022. Throughout this period, the Northeast was the only region consistently above the national average in Medicare Part B–serving psychiatrists per capita.

### Variation in State-Level Participation Rates

**Figure 2.**
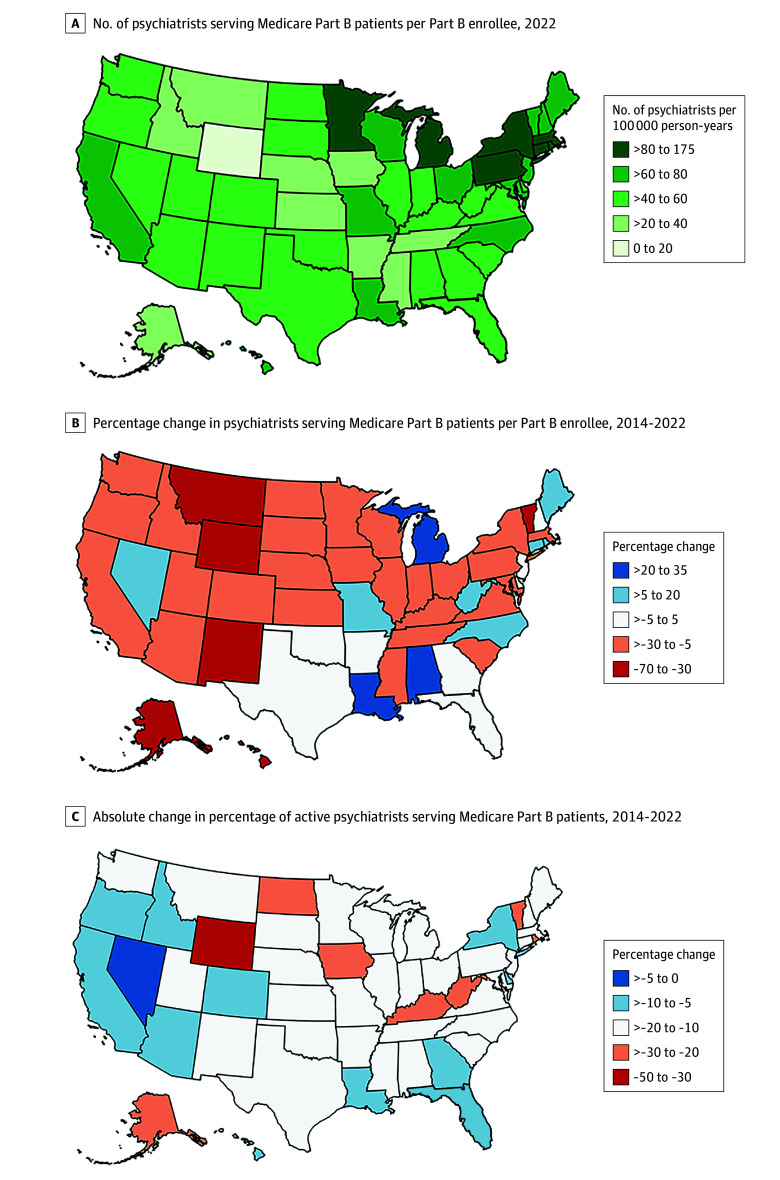
State-Level Distribution of Medicare Part B–Serving Psychiatrists, 2014-2022

In 2022, the highest numbers of traditional Medicare Part B–serving psychiatrists per 100 000 Medicare enrollees were in Rhode Island at 174.7, the District of Columbia at 163.7, and Connecticut at 126.9 (Figure 2A and eTable 3 in Supplement 1). The lowest numbers of Medicare Part B–serving psychiatrists per 100 000 enrollees during that year were in Wyoming at 13.8, Mississippi at 22.1, and Montana at 27.4; Wyoming had less than one-tenth the per-enrollee Medicare-serving psychiatrists than the District of Columbia and Rhode Island. Between 2014 and 2022, the state with the greatest decrease in per-enrollee psychiatrists was also Wyoming, with a reduction of 67.8% (from 42.9 to 13.8 active psychiatrists per 100 000 enrollees; CAGR, −13.2%) (Figure 2B). The state with the largest increase in per-enrollee psychiatrists from 2014 to 2022 was Alabama, with an increase of 31.7% (from 36.5 to 48.1 active psychiatrists per 100 000 enrollees; CAGR, 3.5%).

From 2014 to 2022, the number of traditional Medicare Part B–serving psychiatrists per enrollee decreased for 36 of the 50 states and 1 federal district assessed. In every state and federal district assessed, the percentage of active psychiatrists billing Medicare Part B for professional services decreased from 2014 to 2022 (Figure 2C). The 10 states with the highest proportion of Medicare Advantage enrollees among those enrolled in Medicare in 2021, the year with most recent available total Medicare enrollment data (Minnesota, Michigan, Rhode Island, Florida, Alabama, Hawaii, Wisconsin, Oregon, Ohio, and Connecticut) had declines in their proportion of traditional Medicare Part B–serving psychiatrists (mean [SD] decrease, 27.2% [5.5%]), similar to the 9 states and 1 federal district with the lowest proportion of Medicare Advantage enrollees (Alaska, Wyoming, Maryland, Vermont, North Dakota, Montana, Delaware, Nebraska, District of Columbia, and Kansas; mean [SD] decrease, 34.7% [14.0%]; *P* = .55 for interaction term of group and time).

## Discussion

In this study, we observed a 16.8% decrease in the number of psychiatrists (loss of 3772) billing traditional Medicare for professional services from 2014 to 2022, a period in which the number of professionally active psychiatrists in the US increased by 12.1% (gain of 6076). In this study, there was a per-enrollee decline in Medicare Part B–accepting psychiatrists across every region across the US, despite a more than decade-long increase in the number of medical students entering training to become psychiatrists each year. These findings of decreasing numbers of psychiatrists serving Medicare Part B enrollees despite increasing numbers of these active professionals have, to our knowledge, not been documented for any other medical specialty.

Our results suggest a need for further serious study of (1) potentially long-term diminishing access to psychiatric care for a growing and vulnerable patient population and (2) increased strain placed on the remaining practitioners that accept traditional Medicare. It appears that efforts to increase the psychiatric workforce have not translated to an increase in accessible care for some of the most vulnerable in the US, even as behavioral health crises in this population continue to rise^5^; although the number of professionally active psychiatrists increased by 6076 over the study period, the number submitting more than 10 claims to Medicare Part B for professional services shrank by 3772 psychiatrists during this time. Although one might assume that an increasing psychiatric workforce begets increased access to psychiatric care, our findings suggest that this may not the case for those relying on traditional Medicare to cover costs rather than cash pay or through enrollment in a privately managed Medicare Advantage plan. It may be that the US is entering a period of “paucity in the land of plenty,” where an increased number of psychiatrists does not translate to equitable availability of psychiatrist-led care.^24^ More work is needed to determine whether this longitudinal decline in the proportion of psychiatrists billing traditional Medicare has translated into a difference in the proportion of total Medicare enrollees receiving timely and warranted mental health care. This would require data on Medicare Advantage plans, which serve a similar population but are harder to evaluate because much of their data are proprietary rather than public.^25^

Psychiatrists demonstrate limited acceptance for insurance in general and even less so for government-sponsored options.^24^ In fact, psychiatrists accept Medicare as well as other public and private insurance options at substantially lower rates than physicians of other specialties.^8,9^ This barrier to care presents a challenge for a field of medicine in which (1) accessing care is particularly difficult and (2) those with the most severe illness are more likely to be treated by those accepting insurance, requiring longer and more frequent episodes of care.^26,27,28,29^ We concur with the opinions of previous researchers that low acceptance of insurance among psychiatrists could be attributable to low reimbursements compared with other specialties, a protracted national shortage of psychiatrists enabling insurance-free practice due to nearly unlimited demand, and the regulatory and logistical hurdles of billing Medicare for solo practitioners.^8,9,30,31^

Fewer psychiatrists participate in Medicare Part B each year, as observed in this study. The reasons for this decreased participation in Medicare Part B are likely multifactorial. Further work is warranted to study the causes of these trends, which appear not to be universal across other fields of medicine^32^ and appear unrelated to the traditional or Medicare Advantage coverage mix in a given state, to inform future policy ensuring not only an adequate but also an equitable supply of psychiatrists in terms of geography and the payer status of patients served. Our findings identify states that should command particular concern, states with the steepest declines in Medicare Part B–serving psychiatrists per enrollee, with decreases that suggest potentially drastic losses in access to psychiatric care. These cases may warrant closer investigation as to the current availability of psychiatric care and root causes of such declines, as well as corrective state-level policy action such as recruitment and retention incentives, creation of state-level behavioral health workforce development plans, or tuition reimbursement for those training to become behavioral health care specialists.^33^ Many of the states with the lowest (and highest) concentrations of psychiatrists per capita have comparatively low numbers of traditional Medicare Part B beneficiaries; thus, relatively modest increases in the numbers of practicing psychiatrists accepting traditional Part B in these states could have substantial effects on per-enrollee psychiatrist availability.

Because mental health parity is now present in traditional Medicare Part B^28^ but is not mandated for Medicare Advantage plans (Part C), it would be optimistic to assume that shortages in psychiatric care under the former have been compensated for by expansions in coverage in the latter. A route to expanding mental health access across Medicare currently under discussion among legislators in Washington, DC, is to extend parity protections to Part C, which could help older adults and individuals with disabilities to have access to psychiatric care regardless of which Medicare arrangement they prefer.^34^

### Limitations

This study’s findings should be considered within the context of several limitations. Our study is limited to traditional fee-for-service Medicare data, because these data are freely and publicly available to researchers (although access to these data may soon change), and our results should not be conflated to represent the number of psychiatrists accepting Medicare more broadly (especially Medicare Advantage).^35^ Our denominator data are from the Kaiser Family Foundation and limited in scope, which does not allow for further interrogation of active physician characteristics such as age or sex. We did not account for nurse practitioners or physician assistants who may have played a greater role in serving Medicare patients as the number of psychiatrists decreased, as suggested by a 2022 study,^36^ although stratification of available psychiatric practitioner type by insurance status is an equity issue in itself and one that may prove increasingly important in the future. Finally, we do not address the rise of telepsychiatry, which may distort pandemic-year data. Nevertheless, this study is the first, to our knowledge, to characterize the decline in psychiatrists providing professional services to fee-for-service Medicare Part B patients on the national, regional, and state levels, while placing these findings in the context of a workforce that has been long increasing.

## Conclusions

In this repeated cross-sectional study of all traditional Medicare Part B enrollees from 2014 to 2022, we found significant declines in psychiatrists per enrollee, nationally and across all geographic regions. The nationwide proportion of psychiatrists billing Medicare Part B declined during a time when the total number of active psychiatrists increased. We further identified sizable regional and state-level variation in access to care, with the most underserved state having less than one-tenth the per-capita psychiatrists serving Medicare Part B of the most served state. Taken together, the results of this study suggest a potential decline in access to psychiatrist-led care among many individuals with mental and physical disabilities and older adults nationwide. The causes of these findings and their implications on mental health care access and equity merit further research.

## Notes

Supplement 1. **eTable 1.** Descriptive Codes Used to Identify Psychiatrists in Medicare Data **eTable 2.** Regional Trends in Number of Psychiatrists Billing Services Under Medicare Part B per 100 000 Medicare Enrollees and Comparison to National Average, 2014-2022 **eTable 3.** State-by-State Trends in Number of Non-Advantage Medicare Part B–Serving Psychiatrists per 100 000 Part B Enrollees and Comparison to National Average, 2014-2022

Supplement 2.
Data Sharing Statement
