# Supplementary material for: Trends in Psychiatrist-Led Care for Medicare Part B Enrollees
Source: JAMA Netw Open. 2025 Feb 6;8(2):e2458160. doi: 10.1001/jamanetworkopen.2024.58160 (PMC11803478; doi:10.1001/jamanetworkopen.2024.58160)
Supplement: Supplement 1. — eTable 1. Descriptive Codes Used to Identify Psychiatrists in Medicare Data eTable 2. Regional Trends in Number of Psychiatrists Billing Services Under Medicare Part B per 100 000 Medicare Enrollees and Comparison to National Average, 2014-2022 eTable 3. State-by-State Trends in Number of Non-Advantage Medicare Part B–Serving Psychiatrists per 100 000 Part B Enrollees and Comparison to National Average, 2014-2022 [file jamanetwopen-e2458160-s001.pdf]

Supplementary Online Content

Havlik JL, Wahid S, Priest KC, Ososanya L, Igunbor M, Humphreys K. Trends in psychiatrist-led care for Medicare Part B enrollees. *JAMA Netw Open*. 2025;8(2):e2458160. doi:10.1001/jamanetworkopen.2024.58160

eTable 1. Descriptive Codes Used to Identify Psychiatrists in Medicare Data

eTable 2. Regional Trends in Number of Psychiatrists Billing Services Under Medicare Part B per 100 000 Medicare Enrollees and Comparison to National Average, 2014-2022

eTable 3. State-by-State Trends in Number of Non-Advantage Medicare Part B–Serving Psychiatrists per 100 000 Part B Enrollees and Comparison to National Average, 2014-2022

This supplementary material has been provided by the authors to give readers additional information about their work.

© 2025 Havlik JL et al. *JAMA Network Open*.

eTable 1. Descriptive Codes Used to Identify Psychiatrists in Medicare Data

**Description**
Psychiatry & Neurology: Psychiatry
Psychiatry & Neurology: Addiction Psychiatry
Psychiatry & Neurology: Behavioral Neurology & Neuropsychiatry
Psychiatry & Neurology: Child & Adolescent Psychiatry
Psychiatry & Neurology: Forensic Psychiatry
Psychiatry & Neurology: Geriatric Psychiatry
Psychiatry & Neurology: Psychosomatic Medicine

© 2025 Havlik JL et al. *JAMA Network Open*.

eTable 2. Regional Trends in Number of Psychiatrists Billing Services Under Medicare Part B per 100 000 Medicare Enrollees and Comparison to National Average, 2014-2022

Region	2014		2022		2014-2022		
	Active Psychiatrists Per 100,000 Medicare Beneficiaries	Comparison with National Average	Active Psychiatrists Per 100,000 Medicare Beneficiaries	Comparison with National Average	CAGR[a]	Provider % Growth	Medicare Beneficiaries % Growth
Midwest	63.9	-4.3	61.2	-1.9	-0.5	-18.8	-15.2
Northeast	108.8	40.6	100.0	36.9	-1.0	-19.1	-12.0
South	52.4	-15.9	50.9	-12.2	-0.4	-15.7	-13.3
West	65.3	-3.0	55.7	-7.4	-2.0	-12.7	2.2
National	67.8	-	63.1	-	-0.90	-16.8	-10.6

[a]CAGR: Compound annual growth rate.

© 2025 Havlik JL et al. *JAMA Network Open*.

eTable 3. State-by-State Trends in Number of Non-Advantage Medicare Part B–Serving Psychiatrists per 100 000 Part B Enrollees and Comparison to National Average, 2014-2022

Statistic	Psychiatrists Per 100,000 Part B Medicare Enrollees		Psychiatrists Per 100,000 Part B Medicare Enrollees		Percent change		
**Time period**	**2014**		**2022**		**2014-2022**		
**State**	State statistic	Difference versus National Average	State statistic	Comparison with National Average	CAGR[a]	Per Part B Enrollee Providers	Part B Medicare Beneficiaries
AL	36.5	-32.3	48.1	-13.2	3.5	31.7	-36.1
AK	87.7	18.9	37.6	-23.7	-10.1	-57.1	37.7
AZ	55.3	-13.5	45.4	-16.0	-2.5	-18.0	8.0
AR	36.2	-32.6	36.6	-24.8	0.1	1.0	-15.5
CA	73.1	4.3	67.6	6.2	-1.0	-7.5	-1.0
CO	69.6	0.8	59.5	-1.8	-1.9	-14.5	2.1
CT	116.7	47.9	126.9	65.6	1.1	8.8	-32.3
DE	47.2	-21.6	45.7	-15.6	-0.4	-3.3	1.9
DC	186.4	117.6	163.7	102.3	-1.6	-12.2	-13.8
FL	49.5	-19.3	52.0	-9.4	0.6	4.9	-8.1
GA	51.1	-17.7	51.7	-9.6	0.2	1.3	-16.8
HI	99.2	30.4	61.6	0.3	-5.8	-37.9	5.7
ID	35.3	-33.5	33.1	-28.2	-0.8	-6.3	10.3
IL	57.2	-11.6	53.4	-7.9	-0.9	-6.7	-17.5
IN	47.0	-21.8	42.7	-18.6	-1.2	-9.1	-17.9
IA	40.8	-28.0	37.7	-23.6	-1.0	-7.5	-8.0
KS	46.3	-22.5	39.8	-21.6	-1.9	-14.1	-4.8
KY	47.4	-21.4	44.8	-16.5	-0.7	-5.4	-25.5
LA	50.7	-18.1	61.2	-0.1	2.4	20.6	-22.0
ME	68.7	-0.1	74.1	12.8	0.9	7.8	-30.9
MD	88.6	19.8	63.8	2.5	-4.0	-28.0	3.8
MA	147.3	78.5	119.3	58.0	-2.6	-19.0	-0.9
MI	67.6	-1.2	82.1	20.8	2.5	21.4	-30.2
MN	119.8	51.0	98.1	36.8	-2.5	-18.2	12.7

© 2025 Havlik JL et al. *JAMA Network Open*.

MS	26.8	-42.0	22.1	-39.2	-2.4	-17.5	-17.5
MO	63.1	-5.7	67.5	6.2	0.9	7.0	-21.2
MT	41.4	-27.4	27.4	-33.9	-5.0	-33.9	14.1
NE	42.0	-26.8	38.9	-22.4	-0.9	-7.3	-4.7
NV	42.1	-26.7	47.7	-13.7	1.6	13.1	1.0
NH	64.9	-3.9	66.7	5.4	0.3	2.8	-7.0
NJ	72.5	3.7	73.2	11.9	0.1	1.0	-19.4
NM	62.2	-6.6	43.1	-18.2	-4.5	-30.7	-5.5
NY	127.9	59.1	117.2	55.9	-1.1	-8.4	-10.7
NC	60.8	-8.0	64.9	3.6	0.8	6.8	-15.3
ND	77.7	8.9	54.6	-6.8	-4.3	-29.8	1.8
OH	74.6	5.8	68.2	6.9	-1.1	-8.6	-12.7
OK	42.2	-26.6	40.5	-20.8	-0.5	-3.8	-12.7
OR	62.3	-6.5	51.1	-10.3	-2.4	-18.0	5.5
PA	95.6	26.8	83.8	22.5	-1.6	-12.3	-5.8
RI	165.9	97.1	174.7	113.4	0.7	5.3	-22.7
SC	53.6	-15.2	47.4	-13.9	-1.5	-11.5	-5.0
SD	52.0	-16.8	44.1	-17.2	-2.0	-15.2	2.2
TN	44.8	-24.0	38.4	-23.0	-1.9	-14.4	-13.2
TX	52.4	-16.4	50.1	-11.2	-0.5	-4.3	-13.6
UT	65.2	-3.6	55.7	-5.6	-1.9	-14.6	3.0
VT	101.7	32.9	69.0	7.7	-4.7	-32.1	-4.9
VA	59.4	-9.4	50.5	-10.8	-2.0	-15.0	-5.4
WA	57.5	-11.3	44.4	-16.9	-3.2	-22.7	1.0
WV	52.3	-16.5	57.3	-4.0	1.2	9.6	-26.1
WI	79.9	11.1	68.4	7.0	-1.9	-14.4	-12.1
WY	42.9	-25.9	13.8	-47.5	-13.2	-67.8	24.0
**AVG[b]**	67.8	-	63.1	-	-0.9	-7.0	-10.6
**STD[c]**	33.5	33.5	31.5	31.5	2.8	18.0	14.4
**MIN**	26.8	-42.0	13.8	-47.5	-13.2	-67.8	-36.1
**MAX**	186.4	117.6	174.7	113.4	3.5	31.7	37.7

[a]CAGR: Compound Annual Growth Rate; [b]AVG: national average psychiatrists per 100,000 non-Advantage Part B Medicare enrollees; [c]STD: standard deviation of state-level values.

© 2025 Havlik JL et al. *JAMA Network Open*.

© 2025 Havlik JL et al. *JAMA Network Open*.
