# Supplementary material for: Trends in Psychiatrist-Led Care for Medicare Part B Enrollees
Source: JAMA Netw Open. 2025 Feb 6;8(2):e2458160. doi: 10.1001/jamanetworkopen.2024.58160 (PMC11803478; doi:10.1001/jamanetworkopen.2024.58160)
Supplement: Supplement 2. — Data Sharing Statement [file jamanetwopen-e2458160-s002.pdf]

Data Sharing Statement

Havlik. Trends in Psychiatrist-Led Care for Medicare Part B Enrollees. *JAMA Netw Open*. Published February 06, 2025. doi:10.1001/jamanetworkopen.2024.58160

Data
Data available: No

Additional Information
Explanation for why data not available: Data are publicly available through the Centers for Medicare & Medicaid Services and the Kaiser Family Foundation.
